# Refugee students' perspectives on inclusive and exclusive school experiences in Austria

**DOI:** 10.1002/ijop.12662

**Published:** 2020-02-03

**Authors:** Edvina Bešić, Barbara Gasteiger‐Klicpera, Claudia Buchart, Jessica Hafner, Elisabeth Stefitz

**Affiliations:** ^1^ Institute of Professional Development in Education University of Graz Graz Austria

**Keywords:** Refugee students, School connectedness, Friendships, Social exclusion, Social support

## Abstract

This paper examines refugee students' experiences in the Austrian mainstream school system. It highlights four areas: school connectedness, social exclusion, support systems and friendships. In the study, 55 refugee students between 8 and 21 years old enrolled in primary and secondary education participated in a semi‐structured interview. Data were analysed with directed qualitative content analysis, whereby codes were created deductively and inductively. Students stressed the importance of schooling in order to prosper in the future, particularly through language acquisition. Peers and bilingual teachers played an important role in their efforts to learn German and develop feelings of belonging in the school system. While language acquisition was important for the students, they indicated that other support measures (i.e., remedial education) were largely absent. Further, half of the students reported bullying experiences (verbal, social and physical) associated with their refugee status, language proficiency and religious affiliation. This study has implications for school professionals. The scope of support refugee students receive at school must be broadened, forced migration should be addressed in school in order to counteract negative effects of bullying students receive due to their refugee status and school connectedness can be promoted by hiring staff from diverse cultural backgrounds.

Between 2014 and 2015 applications for asylum tripled in Austria (from 28,064 to 88,340) (BM.I, 2015), which has led to anti‐immigration and anti‐asylum rhetoric dominating domestic politics (Scheibelhofer, 2017). Since families filed many of these applications, 18,468 refugee children were enrolled in Austria's school system by June 2017 (BMB, 2017). Schools are one of the first and most influential service systems that young refugees encounter in the country of settlement. This contact between school and child, where a sense of school connectedness can develop, is crucial “for the successful relocation of refugee children” (McBrien, 2005, p. 337).

School connectedness is seen as a multidimensional concept manifested at different levels. These include an emotional (i.e., feeling safe, supported and valued by teachers and peers), behavioural (i.e., engagement with school activities) and cognitive level (i.e., faith in school values and its significance) (Khawaja, Allan, & Schweitzer, 2018). School connectedness is considered to have a number of positive outcomes for students, including protecting students' mental health, enhancing self‐esteem and efficacy and improving wellbeing (Fazel, Garcia, & Stein, 2016). It also promotes social inclusion, encourages resilience and potential and opens future avenues for success relating to education and job prospects (Block, Cross, Riggs, & Gibbs, 2014).

Teachers and peers play an important role in school connectedness and can foster feelings of belonging. Teachers, on the one hand, can promote positive cross‐cultural socialising and help students develop social skills to ease interactions with peers. Peer relationships, on the other, are associated with improved psychological functioning (Fazel et al., 2016) and offer refugee students a network of support, which helps them cope with social and emotional challenges. Moreover, they act as a “bridge” between the host country and the recent arrival, particularly in the area of language learning (Madziva & Thondhlana, 2017).

Peer interactions, however, can also have negative effects, such as bullying. Exposure to bullying has been associated with a wide range of negative outcomes, including psychological distress and negative school outcomes (Inchley et al., 2016). In Austria, bullying affects about one in 10 students, with verbal bullying being more prevalent than physical or social bullying. Students with a migrant background^1^ state their national origin or native language as causes for bullying (Sauer & Ajanovic, 2012).

Since bullies fall back on stereotypes about students' respective countries of origin and/or ethnicities, the concept of Othering—the “process through which a person or group is turned into somebody different from us, an ‘other’ from whom it is possible to distance ourselves” (Wuthnow, 2017, p. 258)—is a useful lens through which to understand these interactions. By drawing a strict boundary between groups, the marginalised group—in this case, refugee students—is deemed not only distant, but also inferior, leading to their stigmatisation, discrimination and eventual exclusion. While Othering and ethnic discrimination often occur amongst peers, teachers can also be perpetrators of this. In their explorative qualitative study of German young adults with Turkish backgrounds, for example, Moffit, Juang, and Syed (2019) recently showed that these students experienced ethnic discrimination from their teachers during their *Gymnasium* (university‐track school) years. Their findings reveal that particular teachers perpetuate discriminatory norms (e.g., no intervention for peer ethnic discrimination), hold low expectations for the students in question and, in this context, promote ethno‐cultural norms of what it means to be “German.” This ethnic discrimination has many consequences on the individual student, lowering self‐esteem, leading to feelings of loneliness and depression and affecting academic performance (Fazel et al., 2016). It can also impede one's sense of belonging within the school community (Schachner, Van de Vijver, & Noack, 2018). While ethnic discrimination is a major risk factor for students' wellbeing and educational outcomes, school connectedness can act as a protective factor (Khawaja, Ibrahim, & Schweitzer, 2017).

In addition to school connectedness, scholars have also found that social support—instrumental and emotional support—enhances refugee students' integration process, protecting students from mental health issues and helping their psychological adjustment to the new environment (Khawaja et al., 2018). In Austrian schools, instrumental support emphasises language acquisition. For students with German as a second language, schools offer additional language courses, literacy classes and lessons in native languages. Students who enter school with little to no knowledge of German, moreover, can be classified for up to 2 years as “non‐regular students,” or students who attend class and do course work but are not graded (Luciak & Biewer, 2011). While schools are crucial environments that can either help or hinder the successful inclusion of refugee students, little scholarship explores how schools can actually support their inclusion process (Block et al., 2014).

This article, highlighting refugee students' (henceforth students') experiences within the school context, aims to fill in this gap. Stemming from a larger project about social–emotional development and inclusive schooling of refugee students, this article examines school connectedness and social support. By doing so, not only can a better understanding of refugee students' needs be gained, but useful educational support strategies can also be identified.

## METHOD

### Participants

The study sample consisted of 55 students (31 girls; 24 boys), between 8 and 21 years of age. One person was older than 17 and enrolled in secondary school. The average age was 12.05 years old.

We used purposive sampling and selected participants according to the following criteria: (a) students with a refugee experience—that is, students who were forced to flee their home country for various reasons—enrolled in the school system; and (b) students with sufficient knowledge of German to be interviewed, which ensured that the interviewees could fully express their views.

Most students were from Iran (*n* = 16) and Afghanistan (*n* = 16), followed by Iraq (*n* = 9), Chechnya (*n* = 9), Turkey (*n* = 2), Mongolia (*n* = 2) and Somalia (*n* = 1). Out of these 55 students, 36 lived in Graz, while the rest lived either in Graz's suburbs or in rural areas of Styria. Students from Graz attended schools with a high proportion of students with migrant backgrounds (see Table 1 for detail).

**Table 1:** Descriptive statistic of students' school type, school location, gender and age range

	Students in city schools (*n* = 36)			Students in suburban and rural schools (*n* = 19)		
School type	*N*	Gender (female)	Age range	*N*	Gender (female)	Age range
Primary school	11	4	8–11	7	4	8–11
New middle school	22	12	11–14	7	3	12–16
Academic secondary school upper cycle	1	1	14	3	3	15, 16, 21
School for intermediate vocational education	2	2	16	2	2	15, 17

### Researchers

The research team consisted of five white female researchers (one senior researcher, who is also a clinical psychologist, and four junior researchers). Two researchers are migrants, one being a Muslim and (former) refugee. This background proved helpful during the project, particularly when developing the interview questions. The two migrant researchers did not conduct the interviews. While we were aware of the advantages this “insider knowledge” could have brought to the project, it could have also blurred the boundaries of effective research. To avoid participants withholding information they might assume to be obvious to the two migrant researchers, we followed Berger's (2015) framework, where those conducting the interviews were “ignorant” and the students were the experts. This also allowed the process to be an empowering experience for the students.

### Recruitment procedure

An international Catholic social organisation was the gatekeeper. The employees met with possible interviewees and their families not only to discuss the project, but also to have participants understand and sign a consent declaration. It was clarified to the students and their families that they could withdraw from the study at any time without a reason. The employees, as persons of trust, explained to the students before the interviews who the research team was and what kind of questions would be asked. They also spoke with the students after the interviews, providing them the opportunity to express their feelings and, if needed, to help them cope with the experience.

### Interview procedure

All interviews were conducted by three junior researchers between January and June 2018. The interviews were held either at the students' home (*n* = 35), in the centre they were temporarily living in (*n* = 17) or in a centre that offered free afternoon care for students between 6 and 15 years of age (*n* = 3). Interviews lasted between 15 and 61 minutes. The average was 29 minutes.

Each interview started with an overview of the interview procedure and an introduction by the interviewer. The interviews ended with a conversation about wishes for the future to ensure that the students left the interview on a positive note.

Due to spatial constraints, parents, siblings or caregivers were in hearing distance for 22 of the interviews. We are aware that this could have been both enabling and constraining, since it may have shaped what the student felt they could say. It is however worth mentioning that the researchers were cordially received at students' homes, and the parents stressed repeatedly that it was important for them that someone was listening to their children's school experience.

### Instrument

For the interviews, a semi‐structured guide was adapted from existing material (see Rauer & Schuck, 2003; Venetz, Zurbriggen, & Eckhart, 2014). The interview questions that specially relate to the responses considered in this article were: “Remember your first day at school, how did students/teachers welcome you?”; “How do you feel in school?”; “What type of support are you receiving at school?”; “Do you have friends at school?”

### Data analysis

All interviews were audio‐recorded, transcribed, coded and analysed through directed qualitative content analysis (Flick, 2014) using MaxQDA. We used deductive coding and created a coding list with explanations of the codes before analysis began. The junior researchers coded the data. During the analysis, additional codes were developed and discussed by the team. Coding conflicts were resolved in a feedback loop between the whole research team after all transcripts had been coded. Four main categories were developed based on the interview guide (school connectedness, social exclusion, friendship and social support). During the coding process, the category “language” was added to the initial categories since it proved important to all categories. The subcategories “victimisation” and “Othering” were added to social exclusion. An excerpt of the codebook can be seen in Table 2 (German quotes were translated by the researchers for publication).

**Table 2:** Codebook excerpt

Category	Description	Coding guideline
School connectedness	Feeling supported and valued by teachers and peers, significance of school, feeling safe	Text related to liking school, reasons for it; initiatives in creating a welcoming culture by teachers and peers
Social exclusion	Descriptions of bullying, loneliness, Othering	Text related to verbal, social and physical bullying, Othering, reasons for it, solution approach
Friendship	Description of friends and friendships	Text related to defining friends and friendships, motives of establishing friendships, teacher's role in making friends
Social support	Providing emotional or instrumental support in school	Text related to receiving support from school stuff and peers

Table 3 provides an example of the coding scheme.

**Table 3:** Analysis example

Meaning unit	Condensed meaning unit	Code	Category
Uh yes, for example, unless I, all the others are going to Italy, so I think next month. I cannot go because I do not have a passport and so on, so I have no Austrian passport. Otherwise I would go, but. I also asked if I could go, even though I'm still in asylum, they said, no, that's not the case, you have to have an Austrian pass first or a two‐day pass (referring to an emergency passport), then you can go, but none of that is possible.	Does not have a passport; cannot do what Austrian passport holders (his classmates) can do; is an asylum seeker; cannot participate in class activity	Excluded, Othered	Social exclusion

## FINDINGS AND DISCUSSION

### Faith in school values, the importance of school and language acquisition

Students in this study had high expectations for education, and saw it as a pathway to future employment and as an initial possibility for a better life. As one student said:

Well, we came from a completely different country, many girls are not allowed to go to school there and there are no possibilities. When I came here, I saw a completely different life and completely new people. And I saw human rights. And then I said “No, I'm allowed to study here and continue learning” and that was a really great thing. (Girl, age 16, Afghanistan).

Most of the students (*n* = 51) reported going to school regularly, that they enjoy school and feel good while there. Schooling, as the students stressed, provided them with opportunities and motivated their engagement with education.

I want to have a good job later and have a good life. That's why it's [going to school] important to me (Girl, age 10, Iraq).

While the students emphasised that schooling provided opportunities later in life, Austrian schools are not fulfilling this task. Rather than acting as the “great equaliser,” Austria's education system reinforces stratification and inequalities between students through its organisational structure. Generally speaking, in the Austrian school system, students with a migrant background, compared to those without, show lower academic achievement, are less likely to enter an upper secondary school and therefore are less likely to start and finish university studies and obtain highly qualified jobs (Bruneforth, Lassnig, Vogtenhuber, Schreiner, & Breit, 2016). While this is often attributed to individual traits, the school system itself is rarely questioned. For example, teachers' judgements play an important role in one's academic path, because students' grades are not the only factor influencing the transition process within Austria's education system, but also teachers' recommendations (Biewer & Luciak, 2011). Research has shown that teachers associate students with migrant backgrounds with negative working habits and thus make less favourable judgments of them, contributing to their overall disadvantage (Glock & Böhmer, 2018).

Nevertheless, another important reason that motivated the interviewees to attend school was the development of their German language abilities.

[I go to school] because, I want to learn German, because I cannot speak German so well. (Girl, age 8, Afghanistan).

Learning German, moreover, was perceived as a skill that not only had to be acquired to effectively function in the school system, but one that would also provide better future opportunities (both economic and social).

Yes, because I can learn German with my friends. I must learn a lot [of German], because I want to go to this one particular school next year. Because that is good for me. (Boy, age 14, Iran).

Yet students also gave other reasons for wanting to learn German that were not only connected to economic gain. In particular, since students expressed feelings of loneliness, they often connected the need to learn German with a desire to develop friendships in school. More than half (*n* = 28) of the students reported that not speaking German was the main reason for their loneliness. One student remarked:

It was difficult…if you cannot speak much German, then you can only talk to those who speak your language. (Girl, age 16, Chechnya).

### Peer and teacher relationships

Loneliness, however, can be overcome through social interactions with peers (Madziva & Thondhlana, 2017). More than half of the students stated that their classmates and friends were important support networks within schools. Friends were important in order to play, not be alone, spend breaks together, share secrets and talk about topics that could not be discussed with family. Most importantly, friends could help a student study and learn German, as the following quote exemplifies:

I have friends with whom I can speak German and learn German. That is good for me. (Boy, age 14, Iran).

As this quote shows, it is difficult to untangle German language acquisition from the development of friendship. Half of the students stated that it was hard to find friends, mainly due to their inability to speak German and refugee status. The students explained that developing their language skills was crucial for these interactions to occur. Yet all interviewees emphasised the importance of having friends, particularly German‐speaking ones, “because,” as one student said, “I can speak with them German, go for a walk and so on.” (Boy, age 14, Iran).

Besides German native speakers, students from the city reported that peers with the same native language were also important because they acted as translators during the initial stages of schooling and helped the students understand their environment:

[At the beginning of schooling] I felt very bad, but my Chechen friend helped me understand German. He translated for me. (Boy, 14, Chechnya).

Peers then, as Madziva and Thondhlana (2017) also found, were used as “resource[s] for supporting the newcomers” (p. 954). However, it is not clear whether this was encouraged by the teachers or initiated by the students.

Similarly, half of the students also reported that having a teacher speak their native language was helpful in order to settle into the new school.

Our teacher was very nice and friendly… so on the first day at school, she came to me and showed me the school. That was a Persian woman who understood me, and I understood her…She showed me everything. (Girl, age 15, Iran).

This quote demonstrates the importance of bilingual teachers in schools in order to ensure well‐equipped support for students whose first language is not German. This result echoes Madziva and Thondhlanas' (2017) argument that with the increase of linguistically diverse students, schools must seriously consider employing bilingual staff to help improve students' school experience. This goal might be difficult to achieve in Austria, however, since most teachers are not bilingual, and students who are bilingual rarely attend university in order to become teachers often due to the structure of the school system and ethnic discrimination in Austria's labour market (see Verwiebe, Seewann, Wolf, & Hacioglu, 2016). A short‐term solution could be to employ bilingual teaching assistants (TA). TAs were very helpful at school (both academic and social support):

There is a [TA] from a boy in our class and she always helps me. Whenever this teacher is not around, I feel so alone, that no one can help me. (Boy, 12 years, Turkey).

Since TAs in Austria must not have specific qualifications to work in school, they could at least provide help during the initial stages of schooling. While TAs are legally responsible to provide support for students identified as having special educational needs, recent research in Austria revealed that schools actually use TAs flexibly (i.e. where and whenever additional support is needed) (Bešić, Paleczek, Krammer, & Gasteiger‐Klicpera, 2017), which opens the possibility that they could be used to assist refugee students as well.

The students also stressed the importance of teacher support. In our study, teachers helped the students settle into the new school environment. They provided language support by either explaining unknown words—“If you did not know the word, for example ‘glass’, then our teacher drew it on the blackboard” (Boy, age 11, Afghanistan)—or tasks in a simple manner and encouraged collaborative learning between students. Schools in the rural area also pooled resources by involving retired teachers. These teachers met students twice a week and either helped with learning German or homework. Most of the students, except for four, stressed that teachers were encouraging and helped improve their self‐efficacy:

This teacher was very good… she helped me… she wanted me to have good grades, she always said you can do it… (Girl, age 16, Chechnya).

The teachers also organised various introductory games on the first day of school, and asked peers to show their new classmates the school, to talk to them so they could learn German quicker, spend breaks together and provide help regarding school issues.

On my first day of school…we had a ball, and once you had the ball you had to say your name and how old you are, then all children had to repeat that. If you did not understand what to do, the teacher would help. She said: “If you don't understand something, you have friends in class who will help you.” The teacher gave [me] a “lucky pig” in a small bag. (Girl, age 12, Chechnya).

Some students also mentioned that they were responsible for specific tasks in the classroom, which also helped them interact with classmates, learn German and increase their feeling of belonging.

On the second day, it was still hard for me to remember the names, but then the teacher gave me the exercise books to hand out to the other students so that I could remember the names more easily…That's why I always handed out the books…(Boy, age 10, Somalia).

While these strategies from teachers might seem superficial, the students described them as incredibly helpful. In contrast to Albrecht and Ko's (2017) study that found high levels of social support in Canadian secondary schools, it was mainly primary school teachers who initiated these actions in our study.

In the realm of social support, instrumental support (German acquisition and homework) was provided to 11 interviewees during afternoon care at school. This was important for the students since according to them, their parents could not help due to language barriers. These students also stressed that it was beneficial for them when teachers used PowerPoint because it helped them learn German and simultaneously follow instructions. However, only two students reported being able to use ICTs (information and communications technologies, i.e., computers, smartphones) for translation.

This begs the question as to why more teachers did not use ICTs, especially considering that ICTs enable the shift from teacher‐centred to student‐centred teaching approaches. Furthermore, AbuJarour and Krasnova (2017) found in their study about inclusion of Syrian refugees in German society that refugees rely heavily on technology (especially smartphones) to learn the language and culture of the host country. The Austrian National Education Report states that although Austrian teachers use ICTs to lesson plan, they do not utilise ICTs for teaching and learning because they lack the expertise to do so. Not using ICTs with refugee students, then, is part of a larger problem relating to lack of teacher knowledge of how to use ICTs in class, which could be remedied through training (Baumgartner, Brandhofer, Ebner, Gradinger, & Korte, 2016).

When asked if the support provided was enough, 34 students felt sufficiently supported and 15 thought otherwise (all in secondary education). In addition to assistance in learning German, they also needed help in other subjects: “You can learn German anywhere [get support], but not English” (Girl, age 15, Chechnya). This result shows that measures targeted at improving the performance of refugee students are conflated with measures aimed at students with a migrant background that are often restricted to selective compensatory arrangements focusing on language assessment and instruction in the second language (Luciak & Biewer, 2011). However, students stressed that other needs were rarely taken into consideration:

They have … I think they have no time. … Some help me. In German, for example, my teacher helps me a lot. But in the other subjects, I'm not helped so much. I am also ashamed when I ask for help and, in that way, that the teaching time away from others. The others cannot understand that. I do not want the others to be annoyed with me or upset. (Girl, age 21, Iran).

This example reveals the need to provide refugee students other types of support outside the realm of language acquisition, particularly since, as this example highlights, the students will not deliberately ask for it. Furthermore, it stresses the need to foster the students' help seeking behaviour.

Although the students described their schooling in Austria as mainly positive, aspects of social exclusion emerged in student's narratives as well.

### Experiences of social exclusion

Out of the 55 students, 25 reported social, verbal and/or psychically bullying. Seven students felt neglected by their classmates, by either being denied a chance to play or being laughed at when not speaking German correctly. Six students in the city described physical bullying. As an 8‐year‐old boy from Afghanistan put it:

The children fought with me. And when we went to school and asked why that is, the teacher does not see that. She says, “It's just that, that's play”. When I hit back then he went to his parents and told them that and then [organisation] came to us and asked us why I hit someone. Then we said that when an Austrian beats me, they do not do anything, but when I ask why I'm beaten, they just say that's just play.

This fits with the findings of Glock and Böhmer (2018), who have shown that particular teachers have more negative implicit attitudes towards students with migrant backgrounds than they do towards students without one. These attitudes, according to the authors, could influence their judgement of students' behaviours, and, in the end, could contribute to disadvantages for students with migrant backgrounds in school. To address this issue, teachers need to be aware of their attitudes and behaviour and what consequences these might have on students. In the case of the students in our study, previous unsuccessful attempts at eliciting help from teachers resulted in students' reluctance to seek help later in the semester. School staff therefore must realise how race and ethnicity are constructed at school in order to create inclusive and respectful learning environments for all students. Teachers need to be aware of their attitudes and behaviour and what consequences these might have on students. In the case of the students in this study, previous unsuccessful attempts at eliciting help from teachers resulted in students' reluctance to seek help later in the semester.

While physical bullying was more widespread in city schools, verbal bullying affected the students equally regardless of school location. According to students, the main reason for this was their refugee status. In one boy's view:

Well, sometimes I get bullied. Some come to me and say “foreigner”. They think we get 800€ every month, but that's not the case. We get 5€ every day. And with that we cannot even (…) buy so much. And that's why some people are annoying. But not all. (Boy, age 10, Afghanistan).

This quote illustrates how refugees are often constructed in current Austrian discourse, namely, that they are illegal migrants, criminals or people who are taking advantage of the social system and threatening Austrian culture and values. This discourse has led to the creation of a media narrative of “economic refugees” whose main interests in Austria are social welfare and unemployment benefits (Scheibelhofer, 2017). The students highlighted the role of the media and noticed that bullying occurred when the media reported on refugees “doing something bad”. According to one student:

My class does not know that every person is different. For example, I am Muslim and another person is, too, and she is bad and I am not, or I am bad and she is nice. There are different people. It makes me sad when someone in school says that a foreigner did something wrong and, for example, a classmate looks at me and says ‘foreigner’. That makes me sad. (Girl, age 17, Iran).

This indicates that the current media discourse about refugees should be discussed in class in order to prevent bullying due a student's refugee status, helping buffer the negative effects this type of discrimination could have on the integration and social inclusion of students in school and society. This could also promote an inclusive environment, one in which all students' voices and perspectives are acknowledged and respected (Taylor & Sidhu, 2012).

In addition to bullying, students also mentioned times they were Othered during school hours.

In the beginning I could not speak German and make contact with others. But I tried to talk to the others in English. Later, when I could speak German, I asked them “how was my first day at school in your eyes?”. They said, “When we saw you, we thought, oh, what's the old woman doing here in school?” On my first day I really looked like an old woman. My classmates then said, “Old women are wearing headscarves, so we thought maybe you're an old woman too. We thought that a 30‐year‐old woman who does not speak German and so on comes to us”. Then they said, “When you took off your headscarf, we thought, there's the girl we expected”. (Girl, age 15, Iran).

Being Othered by her peers based on appearance due to the visible symbol of the headscarf forced this girl to feel outside the mainstream, resulting in her eventual removal of it. According to the European Islamophobia Report (Bayraklı & Hafez, 2017), Muslim schoolgirls in Austria face discrimination due to wearing a hijab. In the example above, it is also important to note how entangled the role of language is with appearance. She was not only excluded due to her appearance, but also because of her perceived inability to speak German *due to this appearance*. In this context, the headscarf acted as a larger signifier for a lack of German knowledge. The visible difference was used to exclude this student.

More than half of the students described similar processes of Othering at school—often based on physical attributes—which led to their exclusion. This ethnic discrimination separated the student from the perceived “norm”. Besides Othering, the quote above also demonstrates the flipside of acculturation: pressure to abandon a cultural identity in order to fit into a new society (Berry & Sabatier, 2010). This pressure revealed itself by the fact that, only at age 15, this student had already adopted the discourse of integration:

When I put my headscarf away, they were all nicer to me. I was really happy that I integrated myself… (Girl, age 15, Iran).

While Berry and Sabatier (2010) contend that integration allows individuals to maintain a degree of cultural integrity and to identify with both the culture of origin and the culture of the host society, the student in question clearly experienced pressure to “conform to a single cultural standard and a single identity” (Guimond, de la Sablonnière, & Nugier, 2014, p. 147), guided by the perpetuated assumption that refugees “should conform to what we do, because ‘we’ know better than ‘they’ do” (p. 148).

In Austria, this societal pressure to remove a hijab has been recently transformed into law. Since May 2019, it has been illegal to wear a hijab in primary schools. For Muslim refugee students in particular—where the practise of religion is considered a factor for wellbeing (as for religious students of other faiths) (Fazel et al., 2016)—it remains to be seen as to how this will affect their integration process. According to McBrien (2005), though, ethnic discrimination not only leads to a higher likelihood of dropping out of school, but it also negatively affects students' mental health. Moreover, research has shown that ethnic discriminatory experiences of Muslim students have led to lower national identification amongst these students (Schachner et al., 2018)—the opposite of what current political aspirations are.

## CONCLUSION

Our findings suggest that the interviewed refugee students have a sense of school connectedness. The students not only enjoyed attending school and have positive feelings towards it (i.e., emotional level of school connectedness), but they also stressed its importance for prospering in life (i.e., cognitive level). This connectedness stems from peer relationships and certain teachers actively engaging with students (establishing peer contact and involving them in activities). Social support from teachers promoted a sense of belonging within the school community. However, this was mainly present in primary education, revealing the need to establish an adequate support system for refugee students in secondary education as well.

Although students in both primary and secondary education stressed the importance of language acquisition support, 15 students emphasised that other measures were often missing. To implement adequate educational support for refugee students, schools need to abandon the perception that refugee students are part of a “homogeneous” migrant group. In reality, within group differences exist: not all students needed additional support in the same subjects, nor did all experience social exclusion.

Half of the interviewees, however, indeed experienced social exclusion. Students highlighted multifaceted aspects of exclusion and teachers' uneven responses towards it, which disrupted feelings of school connectedness. This result underlines the need for teachers to not only identify ethnic discrimination, but to also address it. To do so, culturally responsive training in anti‐bullying programmes and also “teacher reflexivity regarding” (their own) bias and discussion about ethnic discrimination and its implications are needed (Moffitt et al., 2019). As stated at the outset of this article, schools are one of the first and most influential service systems that refugee students encounter in the host county. Due to this, it is critical that this student group is provided a safe environment free from discrimination in order to succeed.

## Endnotes

1 “The term student with [migrant] background refers to students who themselves or whose parents were not born in Austria” (Luciak & Biewer, 2011, p. 23). In schools, this term is mainly used to specify a student's first language and citizenship status.
